# Inactivation of CDK/pRb Pathway Normalizes Survival Pattern of Lymphoblasts Expressing the FTLD-Progranulin Mutation c.709-1G>A

**DOI:** 10.1371/journal.pone.0037057

**Published:** 2012-05-18

**Authors:** Carolina Alquezar, Noemí Esteras, Ainhoa Alzualde, Fermín Moreno, Matilde S. Ayuso, Adolfo López de Munain, Ángeles Martín-Requero

**Affiliations:** 1 Department of Cellular and Molecular Medicine, Centro de Investigaciones Biológicas (CSIC), Madrid, Spain; 2 Neuroscience Area-Institute Biodonostia, San Sebastian, Spain; 3 Department of Neurology, Hospital Donostia, San Sebastian, Spain; 4 CIBER de Enfermedades Neurodegenerativas (CIBERNED), Madrid, Spain; 5 CIBER de Enfermedades Raras (CIBERER), Valencia, Spain; University of Pennsylvania, United States of America

## Abstract

**Background:**

Mutations in the progranulin (PGRN) gene, leading to haploinsufficiency, cause familial frontotemporal lobar degeneration (FTLD-TDP), although the pathogenic mechanism of PGRN deficit is largely unknown. Allelic loss of PGRN was previously shown to increase the activity of cyclin-dependent kinase (CDK) CDK6/pRb pathway in lymphoblasts expressing the c.709-1G>A PGRN mutation. Since members of the CDK family appear to play a role in neurodegenerative disorders and in apoptotic death of neurons subjected to various insults, we investigated the role of CDK6/pRb in cell survival/death mechanisms following serum deprivation.

**Methodology/Principal Findings:**

We performed a comparative study of cell viability after serum withdrawal of established lymphoblastoid cell lines from control and carriers of c.709-1G>A PGRN mutation, asymptomatic and FTLD-TDP diagnosed individuals. Our results suggest that the CDK6/pRb pathway is enhanced in the c.709-1G>A bearing lymphoblasts. Apparently, this feature allows PGRN-deficient cells to escape from serum withdrawal-induced apoptosis by decreasing the activity of executive caspases and lowering the dissipation of mitochondrial membrane potential and the release of cytochrome c from the mitochondria. Inhibitors of CDK6 expression levels like sodium butyrate or the CDK6 activity such as PD332991 were able to restore the vulnerability of lymphoblasts from FTLD-TDP patients to trophic factor withdrawal.

**Conclusion/Significance:**

The use of PGRN-deficient lymphoblasts from FTLD-TDP patients may be a useful model to investigate cell biochemical aspects of this disease**.** It is suggested that CDK6 could be potentially a therapeutic target for the treatment of the FTLD-TDP.

## Introduction

Frontotemporal Lobar Degeneration (FTLD) is the second most common form of cortical dementia in the presenium, accounting for approximately 20% of dementia patients in this age group. In one of the most common forms of FTLD, there are deposits in the affected regions of ubiquitin-immunoreactive bodies containing mainly TDP-43 protein, and thus named FTLD-TDP [1]. FTLD-TDP is characterized by a widespread atrophy largely affecting the frontal, temporal, and parietal lobes with neuronal loss, reactive atrocytosis, and TDP-43 immunoreactive lesions [2]. The latter include neuronal cytoplasmic inclusions (NCI), less frequent oligodendroglial inclusions (GI), neuronal intranuclear inclusions (NII), and dystrophic neurites (DN). Various subtypes of FTLD-TDP have been proposed based on the proportion and distribution of the TDP-43 immunoreactive lesions [3], [4]. A large subset of FTLD-TDP patients has been identified to harbor loss-of-function mutations (including null mutations) in the gene encoding progranulin (PGRN) [5], [6], and a smaller number of mutations in the valosin-containing protein (VCP) gene [7].

The relationship between PGRN deficiency and FTLD is largely unknown. PGRN is a 593 amino acid, 86 kDa cysteine-rich protein containing a signal peptide and 7.5 repeats of highly conserved granulin motifs [8]. PGRN is widely distributed [8], including the central nervous system (CNS) [4], [5]. Previous research has suggested that PGRN may function as an autocrine neuronal growth factor involved in the inflammatory neuronal repair process in the CNS [9]. Recently, the finding that PGRN binds to the tumor necrosis factor receptor (TNFR) has provided a plausible mechanism to explain the anti-inflammatory action of PGRN [10].

PGRN has been reported to promote neuronal survival in culture [11], though the degree of this effect is controversial [12]. Conversely, PGRN-deficient neurons display reduced survival (but only in stressful conditions, e.g. following H_2_O_2_ administration) [13]. Studies utilizing non-neuronal cells suggest that PGRN can influence apoptosis [13]–[16].

We previously described a prevalent ancestral c.709-1G>A mutation related to Basque population [17], [18]. The c.709-1G>A mutation results in null allele, as most of the pathogenic mutations described up to now, suggesting that FTLD in these families results from PGRN haploinsufficiency [5]. In a recent report from this laboratory, we described alterations in cell cycle-related proteins in lymphoblastoid cell lines derived from peripheral blood mononuclear cells (PBMCs) of c.709-1G>A carriers. In particular, we detected enhanced levels and activity of CDK6 [19] associated with increased cell proliferation. These cell cycle disturbances were considered as systemic manifestations of the proposed aberrant cell cycle activation in neurodegenerative disorders including FTD [20], [21]. On the other hand, there is a growing evidence for the involvement of cell cycle CDKs in neurodegenerative disorders and neuronal apoptosis [21]–[23]. Induction of CDKs occurs in vivo in mature adult neurons during focal stroke and kainate-induced excitotoxicity [24], [25]. It is also seen in neuronal cultures deprived of trophic factors or treated with DNA damaging agents [23]. On these grounds, we found interesting to study the influence of PGRN deficiency on the CDK/pRb pathway and cell survival under conditions of serum deprivation. It is well known that a functional trophic factor deficiency in the microenviroment of vulnerable neurons plays a role in the etiopathogeny of neurodegenerative diseases [26], [27].

Increasing evidences have suggested that subsets of biochemical dysfunctions affecting the brain of neurodegenerative diseases patients may also be traced outside the CNS [28]. Therefore peripheral cells, such as fibroblasts or blood lymphocytes have been extensively used in search for useful biomarkers that may correlate with expression and/or progression of the relative disease [29]–[31]. We demonstrated previously the usefulness of Epstein Barr Virus (EBV)-immortalized lymphocytes to study cell survival/death mechanisms in AD [32]–[35]. Since cellular response is not affected by the viral transformation [32]–[36], the lymphoblastoid cell lines resulting from the EBV transformation represent an easy form to obtain unlimited material to study regulatory mechanisms associated to neurodegeneration. In this work, we carried out a comparative analysis of vulnerability to different noxious stimuli in immortalized lymphocytes from control subjects and individuals carrying the PGRN mutation c.709-1G>A, asymptomatic or suffering from FTLD-TDP. Here, we report an increased resistance to serum withdrawal-induced apoptosis in non-neuronal cells carrying the c.709-1G>A PGRN mutation linked to FTLD-TDP. The protective mechanism involves increased CDK6 activity and it is accompanied by decreased caspase activation and lower dissipation of mitochondrial membrane potential. CDK6 inhibitors sensitize PGRN mutation positive cells to serum withdrawal-induced apoptosis. It is suggested that CDK6 can be a therapeutic target for FTLD-TDP patients.

**Table 1:** Cellular response to stress in control and c.709-1G>A PGRN carriers lymphoblasts.

Condition	% of surviving Cells		
	Control	Asymptomatic	FTLD Patients
H_2_O_2_ (100 µM)	58±6	66±4	67±4
2dRib (30 mM)	69±1	67±8	66±4
SW	66±4	95±3*	92±3*

Lymphoblasts from control and c.709-1G>A PGRN carriers were incubated in serum-free RPMI medium for 72 h (SW) or with 10% FBS in the presence of H_2_O_2_ or 2 deoxy Ribose (2dRib) for 24 h. The cells were then counted by Trypan blue dye exclusion or by the MTT methods. Results are expressed as % of the number of cells at day 0, and are the mean±SE of four independent experiments. Statistical difference: *p<0.05 from lymphoblasts from control individuals.

## Results

### Cellular Response to Stress in Control and c.709-1G>A PGRN Carriers Lymphoblasts

We first studied the cellular response to various insults previously known to cause cell death, such as H_2_O_2_, 2-deoxy-D-ribose (2dRib) [44], [45] or serum deprivation, in lymphoblasts from control and c.709-1G>A carriers. As shown in Table 1, the oxidative noxae, H_2_O_2_ or 2dRib induced cell death in control and PGRN deficient cells. A trend towards more resistance to cell death was observed in PGRN deficient cells although there was no statistically significance. In contrast, lymphoblasts carrying the PGRN mutation appear to be resistant to cell death induced by serum withdrawal.

**Figure 1:**
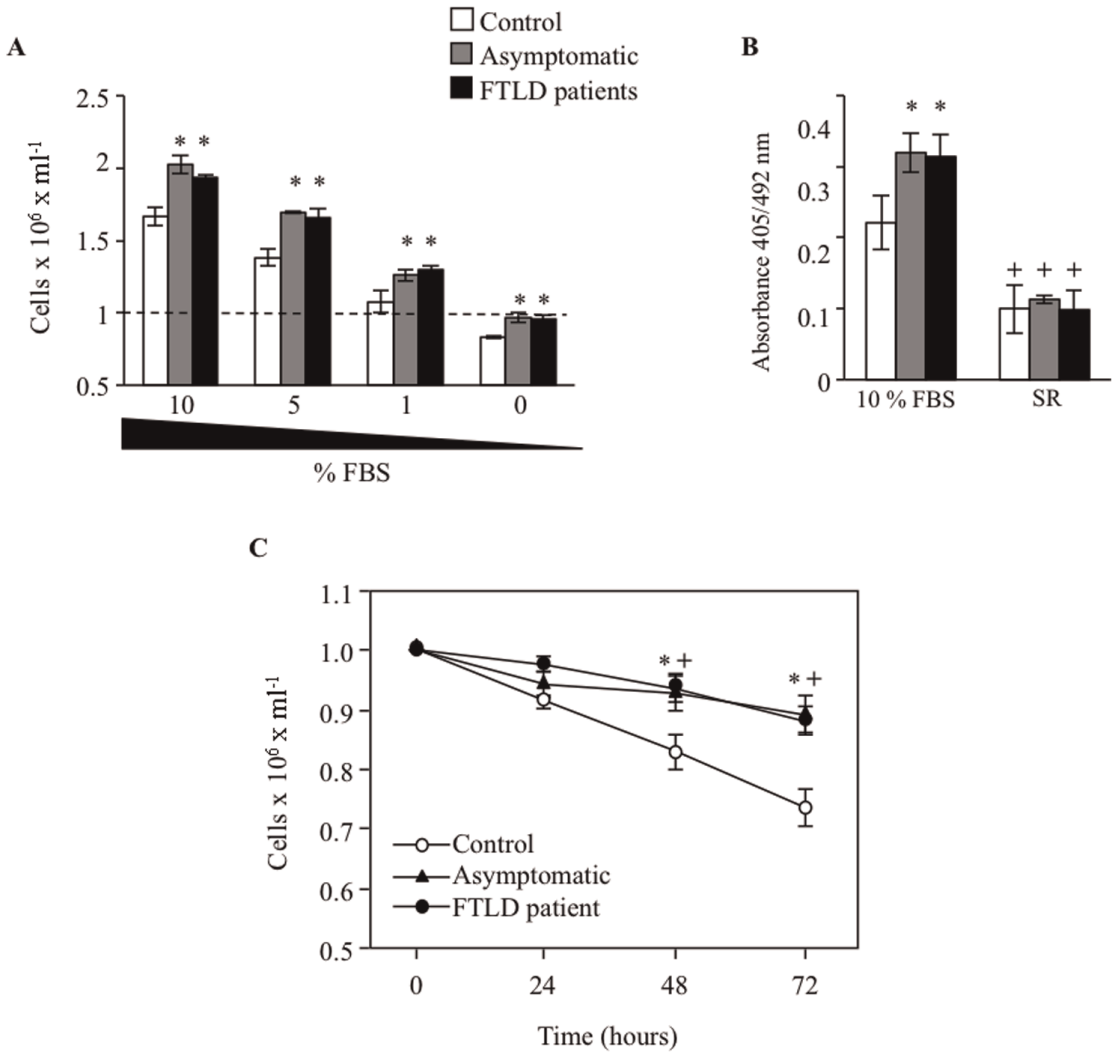
Influence of PGRN haploinsufficiency on cell response to serum stimulation or withdrawal. A: Immortalized lymphocytes from control and c.709-1G>A carriers, FTLD patients or asymptomatic individuals were seeded at an initial density of 1×10^6^/ml and incubated in RPMI medium with decreasing concentrations of FBS or in the absence of serum for 72 h. Cell viability was determined by trypan blue exclusion under inverted phase-contrast microscopy. Data are the mean±SE for at least four independent experiments carried out with cell lines from different individuals *p<0.01 significantly different from control cells. B: Proliferative response of control and PGRN deficient cells in the presence or in the absence of serum. Lymphoblasts (5000 cells/well) were seeded in 96-well plates in the presence of 10% FBS or serum replacement (SR). After 24 h, cells were pulsed with 10 µM BrdU for 4 h. DNA synthesis was assessed by BrdU incorporation method according to the manufacturer’s instructions. Proliferation was expressed as absorbance of stimulated minus that of nonstimulated cultures. Each bar represents the mean±SE of three independent experiments performed in triplicate. C: Effect of PGRN deficiency on the serum deprivation-induced cell death. Lymphoblasts from control and c.709-1G>A carriers, FTLD patients or asymptomatic individuals were seeded as above and incubated in serum-free RPMI medium for 72 h. Cells were harvested every day thereafter and cell viability was determined by trypan blue exclusion under inverted phase-contrast microscopy. Data shown are the mean±SE of all cell lines used in this study (see Table 2). * and ^+^p<0.05 differences significantly different between control and asymptomatic or FTLD patients respectively.

The serum dependence of cell survival for control or PGRN deficient lymphoblasts is shown in Fig. 1A. As expected, the cell number was significantly higher in cultures of c.709-1G>A carriers in the presence of progressively decreasing serum concentrations. This observation is in consonance with previous work demonstrating enhanced proliferative activity in lymphoblasts carrying the PGRN mutation c.7091-G>A [19]. Following 72 h of serum deprivation, the number of cells in control cultures was below the initial seeding while the number of PGRN deficient lymphoblasts did not change. No differences were observed in the proliferative activity (as assessed by BdU incorporation) of control and c.709-1G>A carriers cells under serum replacement (Fig. 1B), thus ruling out that enhanced proliferation could mask the resistance of PGRN deficient cells to serum deprivation-induced death. Data in Fig. 1C summarizes the kinetics of the cellular response to serum deprivation of all cell lines used in this study, derived from carriers of the c.709-1G>A PGRN mutation, asymptomatic and FTLD affected cases, and control individuals. In control cultures, near 30% of cells died after 3-day period of serum starvation, whereas less than 10% of PGRN mutated cells died during the same period of time. It is noteworthy that there was no differences in survival between cells from asymptomatic or patients.

**Table 2:** Demographic characteristics of the subjects enrolled in the study.

	Control n = 10	c.709-1G>A	
		Asymptomaticn = 12	FTLD Patientsn = 7
Age (years)	52±4	53±4	65.4±2.6
Age range	(31–70)	(35–72)	(54–70)
Gender (Male/Female)	5/5	6/6	6/0
PGRN level (range)	95–170	29–61	20–57

Control: individuals without sign of neurological degeneration. Key: c.709-1G>A, progranulin mutation; FTLD, frontotemporal lobar degeneration.

### Serum Withdrawal Induces Apoptosis

**Figure 2:**
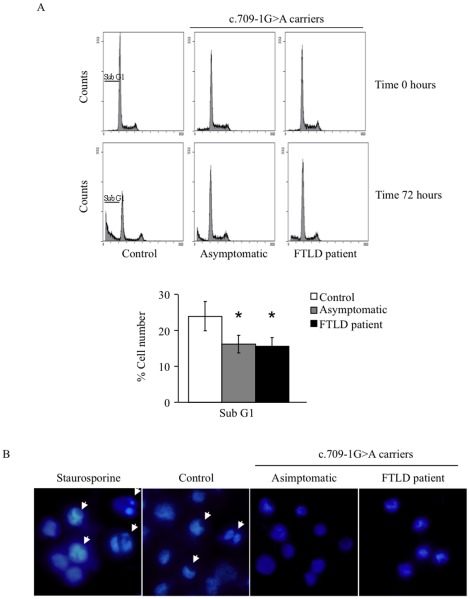
Serum withdrawal induces apoptosis. A: Effect of serum deprivation on distribution of control and c.709-1G>A lymphoblasts in cell cycle. The experimental conditions are identical to those described in the legend of Fig. 1. Cells were harvested before and after 72 h of serum deprivation, fixed and analyzed by flow cytometry as described under Materials and Methods. The percentage of sub-G_0_/G_1_ hypodiploid cells is represented below. Data shown are the mean±SE of different experiments carried out with cell lines from eight control subjects, eight asymptomatic and seven FTLD patients, carrying the PGRN c.709-1 G>A mutation, respectively. *p<0.05 significantly different from control cells. B: Representative photomicrograph showing the presence of chromatin condensation/fragmentation (arrows) in the nuclei of control cells following 72 h of serum withdrawal. As a control of apoptosis, cells from non-demented individuals were treated with 1 µM staurosporine for 5 h. Nuclei were stained with DAPI.

Because cell death can occur via apoptosis or necrosis, it was important to determine which mechanism was involved in serum-starved cells. Apoptosis is characterized by a number of morphological and biochemical events that distinguish it from necrosis. Serum withdrawal-induced cell death was therefore assessed by different procedures. These include 1) flow cytometric analysis of cellular DNA content, 2) microscopic examination of nuclei stained with DAPI, 3) dependence of caspase activity, 4) flow cytometric analysis of mitochondrial membrane potential following serum deprivation, 5) flow cytometric analysis of executive caspases activity by using the Vybrant FAM Caspase-3 and 7 kit (Invitrogen), and 6) assessment of cytochrome c release from the mitochondria. Fig. 2A shows the cell cycle status before and after serum deprivation in control and c.709-1G>A PGRN mutation bearing lymphoblasts. It is shown a higher accumulation of hypodiploid nuclei, following serum withdrawal, in control cultures than in PGRN mutated lymphoblasts. Fig. 2B shows a representative experiment demonstrating chromatin condensation in the nucleus of PGRN mutated cells. As control of apoptosis use was made of staurosporine.

**Figure 3:**
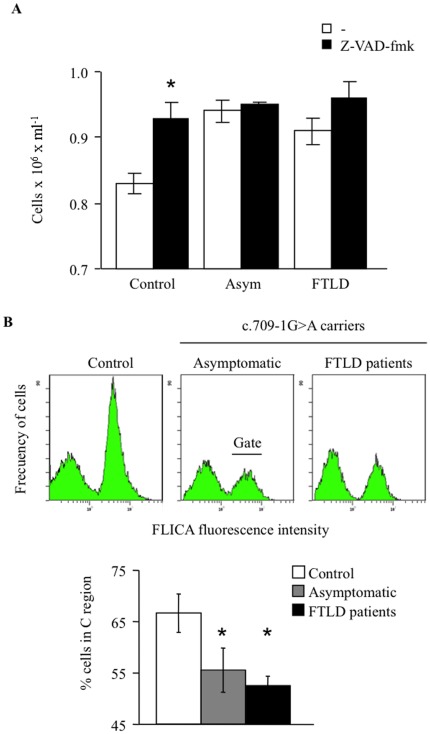
Serum deprivation-induced apoptosis is accompanied by changes in caspase activation. A: Influence of the pan-caspase z-VAD-fmk inhibitor on survival of lymphoblasts derived from control, asymptomatic and FTLD patients following serum deprivation. Cells were seeded at an initial density of 1×10^6^/ml and incubated in serum-free RPMI medium for 72 h in the absence or in the presence of 50 µM z-VAD-fmk for 72 h. Results shown are the mean±SE of different experiments carried out with cell lines from four control subjects, asymptomatic or FTLD patients, carrying the PGRN c.709-1G>A mutation, respectively. *p<0.05 significantly different from control cells. B: Caspase activation in serum-deprived lymphoblasts from control and c.709-1G>A carriers. Cells were incubated as above and then labeled with the FLICA reagent, following the manufacture’s recommendation to detect its binding to active caspases 3 and 7. A representative flow cytometric analysis of the frequency distribution of cells according their green fluorescence is showing. Below it is shown the percentage of cells with active caspases 3 and 7 (mean±SE) of 3 observations carried out in different cell lines from control or c.709-1G>A PGRN mutation carriers individuals. *p<0.05 significantly different from control cells.

To address whether or not the activity of caspases was essential for the observed increase in apoptosis after serum withdrawal, lymphoblasts from control and PGRN mutation carriers were treated with a general caspase inhibitor (z-VAD-fmk). Fig. 3A shows that this compound prevented apoptosis in control cells, without affecting survival of lymphoblasts from c.709-1G>A carriers (either asymptomatic or FTLD patients). The green fluorescent probe FLICA, binds irreversibly to activated caspases 3 and 7, thus increasing the fluorescent signal in apoptotic cells. The assessment of the cell distribution of FLICA fluorescent signal in serum deprived control and PGRN mutated lymphoblasts indicates a higher increase in the activity of executive caspases 3 and 7 in control cells as compared with cells carrying the c.709-1G>A mutation, whether they are asymptomatic or FTLD patients (Fig. 3B).

**Figure 4:**
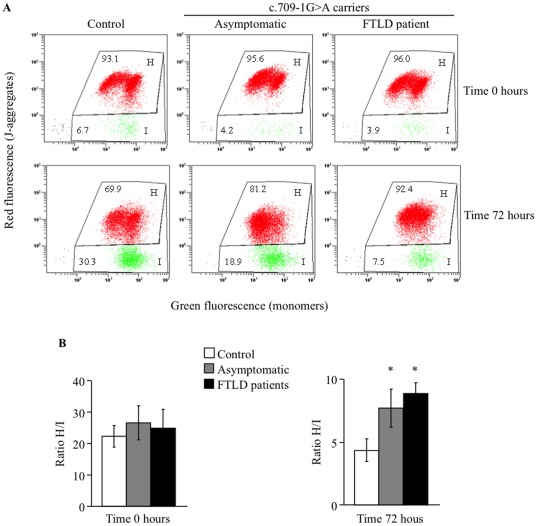
Mitochondrial membrane potential in lymphoblasts from control and c.709-1G>A carrier individuals. A: Lymphoblasts from control and c.709-1G>A carriers (asymptomatic and patients) were labeled with the probe JC-1 following manufacturer’s directions before and 72 h after serum withdrawal. Representative flow cytometric analysis of the frequency distribution of cells according their red or green fluorescence, corresponding to the aggregated or monomeric form of the JC-1 probe is presented. B: The ratio aggregated/monomeric (H/I) form of the JC-1 probe was determined before (left panel) and after 72 hours of serum deprivation (right panel). Values shown are the mean±SE of seven observations carried out in different cell lines from control, asymptomatic or FTLD patients. *p<0.05 significantly different from control cells.

Changes in mitochondrial membrane potential (ΔΦm) were determined by using the fluorescent probe JC-1. After 72 h of serum deprivation, control and c.709-1G>A PGRN mutation carrier cells showed certain degree of mitochondrial membrane depolarization as indicated by a decrease in the red (H)/green (I) JC-1 fluorescence ratio (Fig. 4A). However, there were important differences in the extent of depolarization. After 72 h of serum deprivation (Fig. 4B right panel) the drop in red/green fluorescence ratio was significantly decreased in control cells, while PGRN deficient cells, from asymptomatic or FTLD patient, were only slightly depolarized as expected because of the lack of cell death detected in these conditions. No differences in membrane potential were observed in control and PGRN mutated cells prior to serum starvation (Fig. 4B left panel).

**Figure 5:**
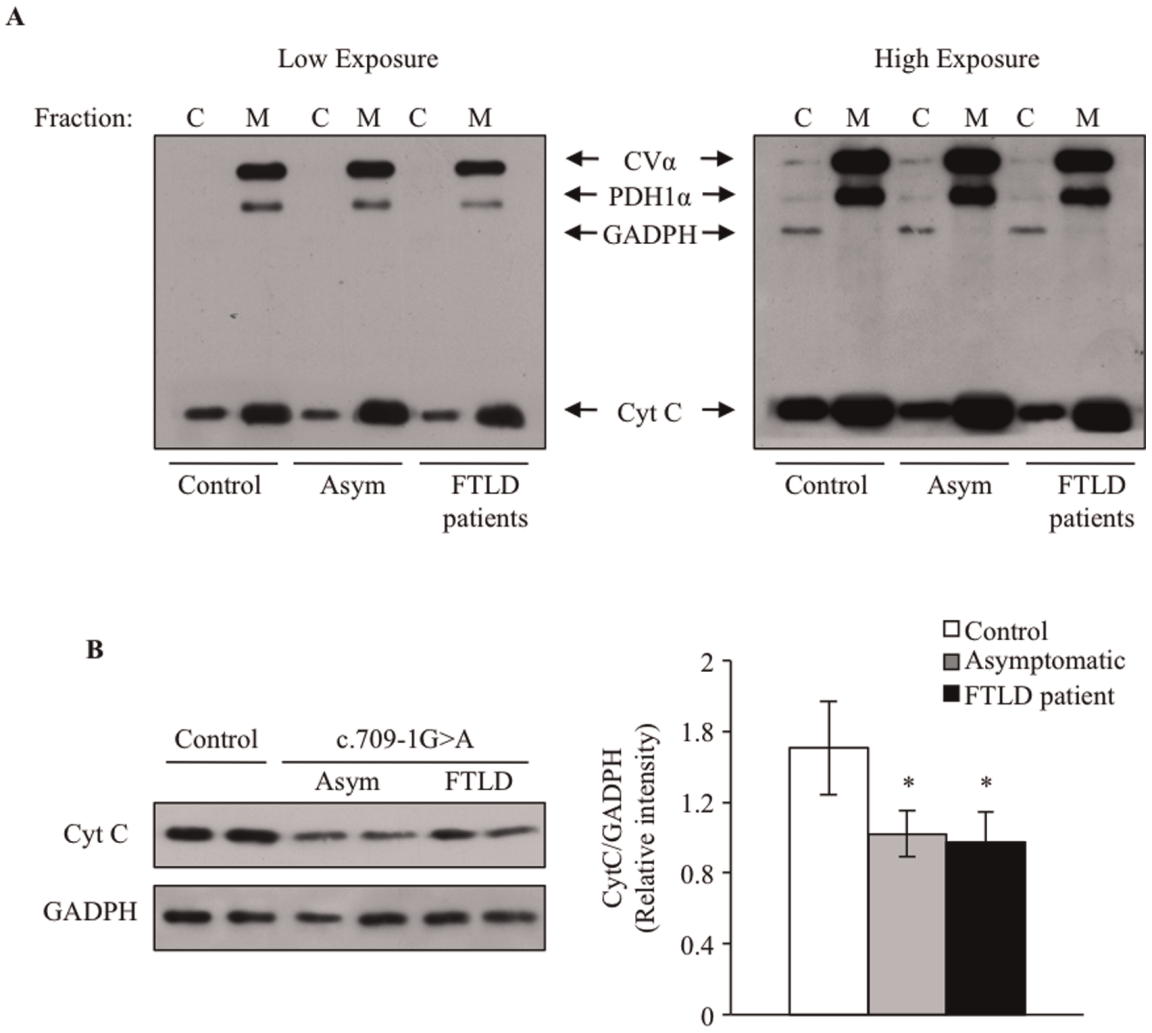
Enhanced release of cytochrome c to the cytosol in serum-deprived lymphoblasts bearing the c.709-1G>A PGRN mutation. A: Lymphoblasts from control and c.709-1G>A carriers were serum deprived for 72 h. Cell lysates were fractionated to isolate cytoplasmic and crude mitochondria. The presence of cytochrome c in cytosolic and mitochondrial fractions was assessed by WB analysis using the ApoTrack antibody cocktail, which demonstrates the purity of the fractions and loading. A representative blot of three independent experiments is shown. B: Cytochorme c detection in cytosolic extracts from control and PGRN deficient lymphoblasts. A representative immunoblot showing cytosolic cytochorme c in two different individuals for each condition is shown (left panel). Densitometric analysis is presented in the right panel. The data represent the mean±SE of the cytosolic cytochrome c for four observations in different cell lines. *p<0.05 significantly different from control cells.

The release of mitochondrial cytochrome c following serum deprivation further indicates the activation of the “intrinsic” (mithochondrial initiated) apoptotic pathway. Fig. 5 shows that serum deprivation-induced release of cytochrome c to the cytosolic compartment is enhanced in control lymphoblasts compared with the c.709-1G>A PGRN mutation bearing cells.

### Role of CDK/pRb Pathway on Cell Survival

**Figure 6:**
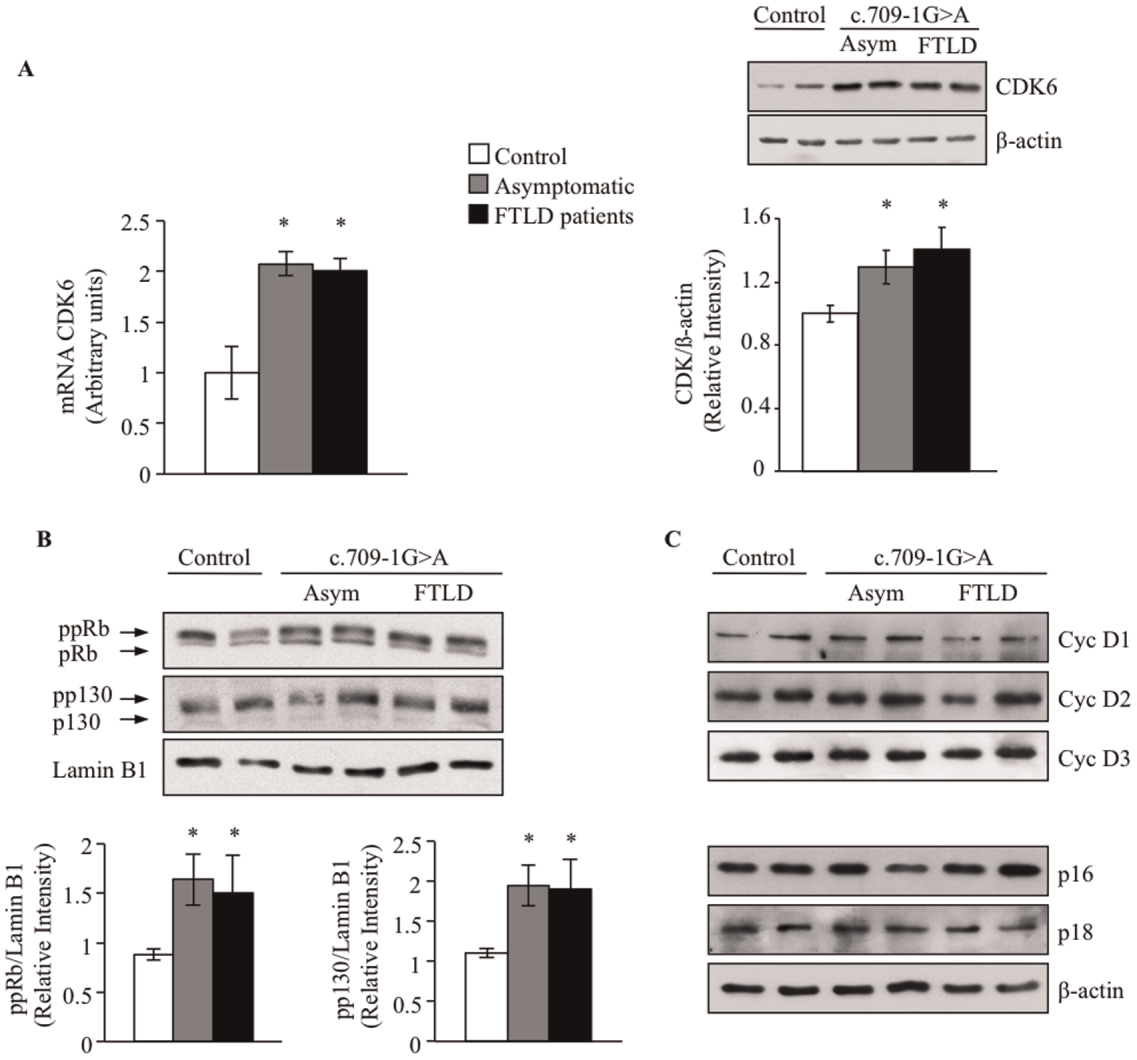
CDK6 mRNA, protein levels and pRb phosphorylation in lymphoblasts from control and c.709-1G>A carriers individuals. Immortalized lymphocytes from control and c.709-1G>A carriers, FTLD patients or asymptomatic individuals were seeded at an initial density of 1×10^6^/ml and incubated in serum-free RPMI medium. 48 hours later cells were harvested to isolate RNA and to prepare cell lysates. A: CDK6 mRNA expression levels were analyzed by quantitative RT-PCR (left panel), and the CDK6 protein content was analyzed by WB (right panel). The inmunoblot shows two different cell extracts from control, asymptomatic and FTLD patients is shown. The data represent the mean±SE for six observations in different cell lines. *p<0.05 significantly different from control cells. B: Representative inmunoblots showing pRb and p130 phosphorylation status in two different control, asymptomatic or FTLD individuals is shown. pp =  the hyperphosphorylated form of the pRb or the p130 protein. Below it is shown the densitometric analysis of the hyperphosphorylated form of pRb and p130. Data represent the mean±SE for six independent observations in different cell lines. C: Representative immunoblots showing the cellular content of Cyclin D1, D2 and D3, and the CDK inhibitors p16 y p18 in two different control, asymptomatic or FTLD individuals.

Previous work from this laboratory indicated that the c.709-1G>A PGRN mutation carriers showed increased activity and levels of CDK6 protein under proliferating conditions [19]. We were interested in evaluating the role of the CDK6/pRb pathway in the survival/death of these cell lines under serum deprivation conditions. First, we determined by quantitative RT-PCR the expression levels of mRNA CDK6 and, by Western blot analysis, the levels of CDK6 and pRb in control and PGRN mutated cells following serum withdrawal. Fig. 6A, shows that both the mRNA levels of CDK6 and protein content increased in PGRN mutated cells incubated in the absence of serum. Taken together our results suggest that increased expression of CDK6 is a distinct feature of PGRN deficient lymphoblasts independent of the presence or absence of serum. CDK6 activity, assessed by pRb and p130 phosphorylation status was increased in c.709-1G>A PGRN mutation carrying cells, either asymptomatic or FLTD patients (Fig. 6B). No differences were found in the levels of cyclins D1, D2 and D3 or in the CDK inhibitors p16 and p18 between control and PGRN-deficient lymphoblasts (Fig. 6C).

**Figure 7:**
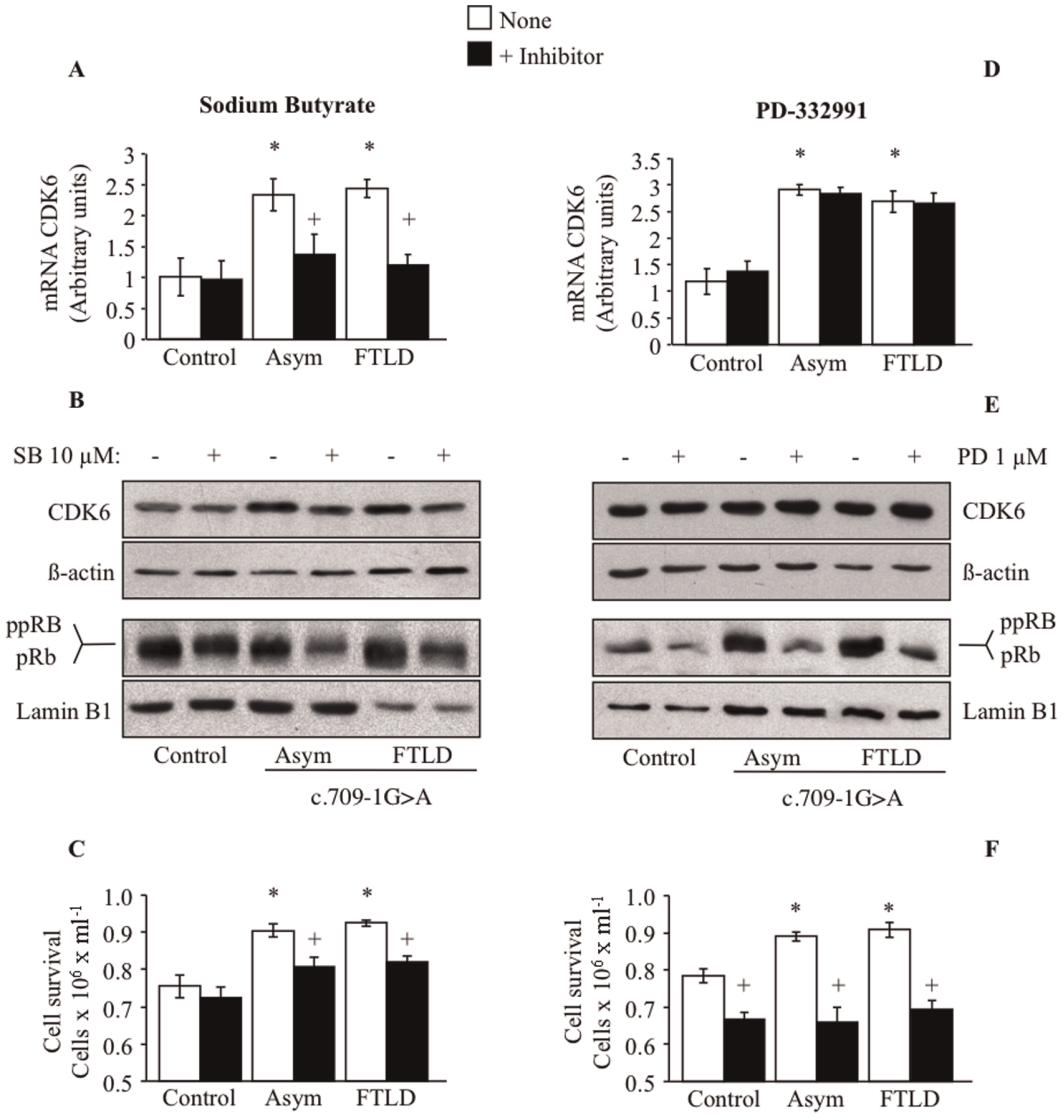
Effects of sodium butyrate and PD332991 on CDK6 mRNA and protein levels and in the survival of control and c.709-1G>A carriers lymphoblasts. Lymphoblasts were incubated as in the legend of Fig. 6 in the absence or in the presence of 10 µM SB (A, B and C) or 1 µM PD332991 (E, F and G) for 48 h. CDK6 mRNA analysis was performed by quantitative RT-PCR, protein levels were assessed by WB. Cell survival was determined by trypan blue exclusion under inverted phase-contrast microscopy. Values shown are the mean±SE for four independent observations carried out in different cell lines. *p<0.05 significantly different from control cells. **p<0.05 significantly different from untreated cells.

We next inhibited CDK6 activity with an inhibitor of histone deacetylases (HDAC) to blunt the CDK6 mRNA expression, such as sodium butyrate (SB). Incubation of cells with SB induced down-regulation of CDK6 mRNA, reduced protein levels and the phosphorylation status of pRb (Fig. 7A,B) and sensitized PGRN mutated cells to serum deprivation-induced cell death (Fig. 7C). Cell survival of control cells was not affected by this dose of SB (10 µM). This dose of SB was proven to be effective in blunting the enhanced proliferative response of PGRN deficient lymphoblasts [19]. On the other hand, we specifically inhibited CDK6 activity with the small molecule PD332991 (Pfizer). We observed that increasing concentrations of this compound (0.5 to 2.5 µM) induced cell death of control and PGRN deficient lymphoblasts in a dose-dependent manner (data not shown). Maximal effects were observed at 1 µM PD332991. Treatment of control and PGRN mutated cells with this dose of PD332991, induced dephosphorylation of pRb protein without changes in the CDK6 mRNA and protein levels in control and PGRN deficient cells (Fig. 7 D y E). This treatment sensitized c.709-1G>A PGRN mutation carrier cells to serum deprivation-induced apoptosis and increased death of control lymphoblasts (Fig. 7F).

### Role of PGRN Haploinsufficiency on the Survival of Immortalized Lymphocytes

**Figure 8:**
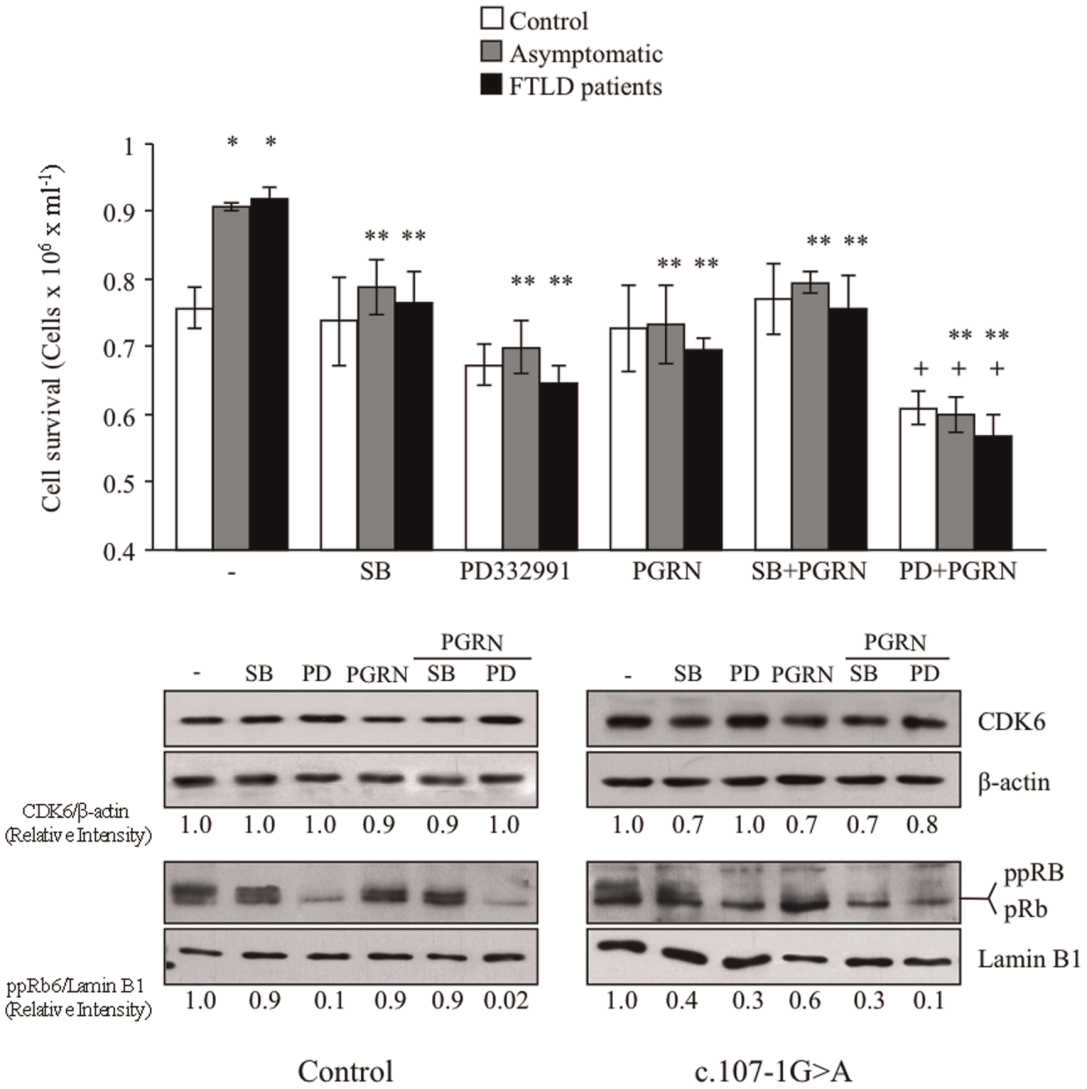
Effects of exogenous progranulin in the serum deprivation-induced cell death. Lymphoblasts from control or c.709-1G>A carriers individuals were incubated in serum-free RPMI medium in the absence or in the presence of recombinant PGRN (100 ng/ml), alone or in combination with 10 µM SB or 1 µM PD332991. Cell survival was determined after 72 hours of serum deprivation. Data shown are the mean±SE of four determinations carried out with different cell lines. *p<0.05 significantly different from control cells. **p<0.05 significantly different from untreated cells. ^+^p<0.05 significantly different from cells treated with PGRN alone. Below it is shown representative immunoblots showing the effects of these drugs, alone or in combination of exogenous progranulin on CDK6 and pRb proteins levels.

To further understand the influence of PGRN deficiency on cell survival, we added recombinant PGRN in the presence or in the absence of CDK6 inhibitors, to the culture medium. Fig. 8 shows that exogenous PGRN mimicked the proapoptotic effects of SB, while cooperated with PD332991 to produce a greater inhibition of phosphorylation of pRb protein and cell survival in PGRN deficient cells. While PGRN or SB did not affect either phosphorylation of pRb or survival of control cells, inhibiting CDK6 activity by PD332991, alone or in combination of PGRN, greatly decreased the phosphorylation of pRb and consequently raised the vulnerability of control cells to serum deprivation. Taken together these results suggest that exogenous PGRN blunted the enhanced CDK6 mRNA expression levels in PGRN deficient cells. Interestingly, the treatment of PGRN deficient cells with exogenous PGRN in the presence of serum had similar effects than SB in preventing the increased proliferative response of these cell lines. Moreover exogenous PGRN and PD332991 showed also additive effects in decreasing cell proliferation (results not shown). These observations highlight the important role of CDK6/pRb pathway in determining the cell fate, survival/death depending on growth factors availability.

## Discussion

Results presented herein indicate that immortalized lymphocytes from c.709-1G>A PGRN mutation carriers, asymptomatic or FLTD patients are more resistant to cell death induced by serum deprivation than those derived from control individuals. These cell lines were previously found to show enhanced proliferative activity upon serum stimulation [19]. These neoplastic-like features of PGRN deficient lymphoblasts may be systemic manifestations of cell cycle-related events associated with neurodegeneration, as described in several diseases of the Central Nervous System (CNS) [46]–[49]. We and others reported previously a selective impairment of mechanisms involved in cell death in peripheral cells from Alzheimer’s disease patients [32], [50]–[52]. Moreover, we demonstrated that the immortalization procedure did not alter the cellular response of fresh obtained lymphocytes, to addition or withdrawal of mitogenic factors [32], [36], thus providing support for considering lymphoblastoid cell lines as suitable model to study cell survival/death mechanisms regulation associated with neurodegeneration and for testing novel modifying-disease therapies.

Cell death induced by serum deprivation showed characteristics of apoptosis. The lower sensitivity of PGRN deficient cells to trophic factors withdrawal was accompanied by lower dissipation of ΔΦm, decreased activation of capases 3 and 7, and reduced cytochrome c release from the mitochondria, compared with control cells.

c.709-1G>A PGRN mutated cells treated with inhibitors of CDK6 activity undergo significant apoptosis in the absence of serum in the culture medium as they do control cells, suggesting a role of the CDK/pRb signaling pathway in protecting PGRN deficient cells from apoptosis. The effects of SB and PD332991 on survival of lymphoblasts bearing the PGRN mutation are in line with the reported effects of PD332991 on myeloma cells inhibiting cell cycle progression and increasing the cell sensitivity to bortezomib-induced apoptosis [53]. Considering that CDK6 inhibitors are already being used for treatment of a number of human tumors [54] with a good tolerance, it is plausible that they may serve as novel therapeutic drugs for FTLD.

The observed enhanced CDK/pRb activity of PGRN deficient lymphoblasts contrasts with the fact that increased CDK activity and pRb phosphorylation have been linked to neuronal death in a number of cell and animal models of neurodegeneration [55], [56]. However, one has to take into account that alteration of cell cycle-related events in brain and lymphocytes have different consequences. Lymphocytes from PGRN mutation carrier individuals show and enhanced proliferative activity [19] and increased resistance to serum deprivation-induced cell death than cells derived from control individuals. On the other hand, cell cycle disturbances in already adult neurons results in cellular dysfunction, premature cell death, and thus neurodegeneration [57]. It is worth mentioning that CDK6 inhibitors addition to PGRN mutated lymphoblasts were able to restore the “normal” cell response to serum stimulation [19] or withdrawal (this manuscript), by blunting the enhanced proliferative activity or sensitizing cells to apoptosis in the absence of serum. In both situations, CDK6 inhibitors decreased the levels of phosphorylation of pRb protein in c.709-1G>A carrier cells to reach those of control cells. It remains to be demonstrated whether CDK6 inhibitors would protect neurons in FTLD brain from apoptosis by modulating the CDK6/pRb pathway, but it has been suggested that SB and other HDAC inhibitors behave as neuroprotective drugs [58]. These compounds prolonged the life span of cultured cortical neurons [59] and promoted neuronal growth. Work carried out in vivo demonstrated that they protected neurons from ischemic stroke [60]. A neuroprotective effect of these drugs has also been reported in animal models of neurodegenerative disorders [61], [62].

The proapoptotic effect of SB in PGRN mutated cells appears to be directly related to PGRN insufficiency since it was mimicked by the addition of recombinant PGRN. The fact that exogenous PGRN mimicked the SB effects but cooperate with the inhibitor of CDK6 activity PD332991 on cell survival, points out to the alteration of CDK6 transcription as the major cause of aberrant cell survival/death response of PGRN deficient cells.

The expression of CDK6 is negatively regulated by TDP-43 [63]. The control of CDK6 expression mediated by TDP-43 involves GT repeats in the target gene sequence. Several reports indicate that PGRN deficiency induced mislocalization of TDP-43 protein leading to a loss of the DNA-RNA binding function of the protein [64], [65]. Indeed an increase in the cytosolic content of TDP-43 protein could be observed in c.709-1G>A bearing lymphoblasts, associated with increased levels of CDK6 [19]. It appears therefore that altered DNA/RNA binding protein function, rather than toxic aggregation is central to TDP-43-related neurodegeneration. Two recent reports support this asseveration, the first one indicates that there is no correlation between protein aggregates formation and severity of the disease [66] while the second suggests that TDP-43 function is required for cell survival in ALS [67].

Our results show no differences in the cellular response to serum deprivation and content of CDK6 among lymphoblasts derived from c.709-1G>A mutation carriers, asymptomatic or presenting already clinical signs of dementia. Since most of the asymptomatic carriers are younger than the patients, this finding suggests that dysfunction of cell survival could be an early manifestation of the disease. Nonetheless, there are c.709-1G>A carriers that remain asymptomatic until advanced age suggesting that the altered cell survival/death response is not sufficient to cause the disease and there must be other genetic or environmental factors in determining the onset of clinical disease.

The reason for the distinct vulnerability to serum deprivation of PGRN deficient cells cannot be ascertained with the present data. However, the possibility should be considered that changes in signaling molecules and/or receptors might be altered. On these grounds, it is worth to mention that it has been recently reported disturbances in circulating levels of several cytokines in the serum of asymptomatic and FLTD patients carriers of loss-of-function PGRN mutations [68]. On the other hand, a recent functional genomic study had revealed changes in Wnt signaling pathway in PGRN deficient cells and demonstrated upregulation of the FZD2 receptor in PGRN knockdown mice [69]. It was suggested FZD2 could play a potentially neuroprotective role in PGRN deficient cells. Moreover, TNF receptor has been identified as a PGRN binding receptor [10]. Therefore, progranulin haploinsufficiency could eventually potentiate TNF-α signaling. Whether similar mechanisms operate in lymphocytes from carriers of c.709-1G>A PGRN mutation is currently under investigation in our laboratory.

Finally, an issue that needs to be taken into account for discussion purposes is that although FTLD associated changes detected in peripheral cells might not fully reflect those in FTLD brain, it is evident that besides neuronal damage there are also peripheral aspects of the disease. A close relationship seems to exist between the state of the immune system, and particularly lymphocytes, and some psychiatry disorders including AD [70]. As far as we know, clinical disturbances in the immune system have not been reported in FTLD. However it is possible that some factors, including neuroinflammatory cytokines that link the peripheral immune and nervous systems can influence neuronal survival in FTLD.

In summary, we provide evidence that CDK6/pRb signaling pathway is enhanced in PGRN deficient cells, associated with altered cell vulnerability to trophic factor deprivation. Exogenous PGRN and inhibitors of CDK6 activity were able to restore the normal cell response. It is suggested that the inhibition of CDK6 activity or alternatively the modulation of PGRN levels may have a beneficial effect on FTLD-TDP. Taken together our results with the recent findings that alkalinizing drugs [71] or the FDA-approved HDAC inhibitor, suberoylanilide hydroxamic acid (SAHA) are able to increase PGRN levels [72], it is possible to envision new promising avenues for therapeutic intervention in FLTD-TPD.

## Materials and Methods

### Materials

All components for cell culture were obtained from Invitrogen (Barcelona, Spain). The kinase inhibitor PD332991 was kindly provided by Pfizer. The inhibitor of histone deacetylases sodium butyrate (SB), 3-(4,5-dimethylthiazol-2-yl)-2,5 diphenyltetrazolium bromide (MTT), 2-Deoxy-D-ribose (2dRib) and H_2_O_2_ were obtained from Sigma-Aldrich. The caspase inhibitor benzyloxy-carbonyl-Val-Asp-fluoromethylketone (z-VAD-fmk) was obtained from Calbiochem (Darmstad, Germany) and 4,6-diamino-2-phenylindole (DAPI) was obtained from Serva (Heidelberg, Germany). Progranulin (human) (recombinant) was obtained from Enzo (Life Sciences). Poly (vinylidene) fluoride (PVDF) membranes for western blots were purchased from Bio-Rad (Richmond, CA). Antibodies against human Cdk6, pRb, p130, p16, p18 were obtained from Santa Cruz Biotechnologies (Santa Cruz, CA). Antibodies against Cyclin D1, D2 and D3 were obtained from Cell Signaling, antibody against Lamin-B1 was obtained from Calbiochem (Darmstad, Germany) and antibody against β-actin was obtained from Sigma-Aldrich. ApoTrack™cytochrome c Apoptotic WB antibody cocktail (ab110415) was obtained for MitoSciences (Eugene, Oregon, US). The enhanced chemiluminiscence (ECL) system was from Amersham (Uppsala, Sweden.). Other reagents were of molecular biology grade.

### Study Samples and Cell Lines

A total of 29 individual were enrolled in this study. We studied 19 individuals with a single pathogenic splicing mutation in the PGRN gene (c.709-1G>A), 7 of them patients of FTLD-TDP, 12 asymptomatic and 10 control individuals without mutation in PGRN nor any sign of neurological degeneration. All patients were of Basque descent. Asymptomatic and control individuals were relatives of patients. All patients were diagnosed as FTD in the Donostia Hospital by applying consensus criteria as published elsewhere [37]. Patients exhibited variable phenotype initial symptoms. Four of them presented the behavioral variant of frontotemporal dementia (bv-FTD), one progressive nonfluent aphasia, and corticobasal basal syndrome (CBS), the other patients developed a relatively rapidly progressive dementia with features that led to a secondary diagnosis. Notably, this secondary diagnosis was CBS in three of the bv-FTD cases. It is shown that PGRN levels in plasma were strongly reduced in affected and unaffected subjects carrying the c.709-1G>A mutation. Table 2 summarizes the demographic characteristics and the plasma levels of PGRN of all subjects enrolled in this study.

All study protocols were approved by the Donostia Hospital and the Spanish Council of Higher Research Institutional Review Board and are in accordance with National and European Union Guidelines. In all cases, peripheral blood samples were taken after written informed consent of the patients or their relatives to determine the presence of the c.709-1G>A PGRN mutation and to establish the lymphoblastoid cell lines.

DNA was extracted from blood cells using standard procedures. PGRN gene sequencing procedures used at our laboratory have been published elsewhere [17]. For determination of PGRN plasma levels we used an ELISA kit (AdipoGene, Korea).

Establishment of lymphoblastoid cell lines was performed in our laboratory as previously described [38], by infecting peripheral blood lymphocytes with the Epstein Barr virus [39]. Cells were grown in suspension in T flasks in an upright position, in approximately 10 ml of RPMI-1640 (Gibco, BRL) medium that contained 2 mM L-glutamine, 100 µg/ml penicillin/streptomycin and, unless otherwise stated, 10% (v/v) fetal bovine serum (FBS) and maintained in a humidified 5% CO_2_ incubator at 37°C. Medium was routinely changed every two days.

### Determination of Cell Proliferation

Cell proliferation was assessed by the 5-bromo-2′-deoxyuridine (BrdU) incorporation method using an enzyme-linked immunoassay kit procured from Roche (Madrid, Spain). Cells (5000 cells/well) were seeded in 96-well microtiter plates. Four hours prior to the end of the interval of measurement, BrdU (10 µM) was added. The cells were fixed with precooled 70% ethanol for 30 min at –20°C and incubated with nucleases following manufacturer’s recommendations. Cells were then treated for 30 min at 37°C with peroxidase-conjugated anti-BrdU antibody. Excess antibody was removed by washing the cells three times, followed by the addition of substrate solution. Absorbance was measured at 405 nm with a reference wavelength of 492 nm.

### Cell Survival Assay

The cell suspension was mixed with a 0.4% (w/v) trypan blue solution and the number of live cells was determined using a hemocytometer. Cells failing to exclude the dye were considered nonviable. In some experiments cell viability was checked by the MTT assay [40] obtaining similar results.

### Assessment of Apoptosis and Caspase Activity

Flow cytometry was performed to determine the content of apoptotic sub-G1 hypo-diploid cells [41]. Exponentially growing cultures of cell lines were seeded at an initial concentration of 1×10^6^ cells/ml and cultured for 72 h in serum-deprived RPMI medium. Then, cells were fixed in 75% ethanol for 1 h at room temperature. Subsequent centrifugation of the samples was followed by incubation of cells in phosphate-buffered saline (PBS) containing 1 µg/ml RNase at room temperature for 20 min and staining with propidium iodide (PI) (25 µg/ml). Cells were analyzed in an EPICS-XL cytofluorimeter (Coulter Científica, Móstoles, Spain). Estimates of cell cycle phase distributions were obtained by computer analysis of DNA content distribution. In addition, apoptosis was characterized by chromatin condensation/fragmentation, as determined by cell permeabilization followed by DAPI staining and microscopy examination.

The activation of executive caspases was investigated using the Vybrant FAM Caspase-3 and 7 Kit (Invitrogen) including FLICA reagent that is retained within the cell, if bound to the active caspase molecule. Lymphoblasts from control and carriers of c.709-1G>A mutation were resuspended in 300 µl of RPMI containing 10 µl of FLICA reagent and incubated in 5% CO_2_ at 37°C for 60 min. The cells were then washed with, and suspended in wash buffer provided with the kit. The samples were analyzed on the flow cytometer.

### Analysis of Mitochondrial Membrane Potential

Changes in mitochondrial membrane potential (ΔΦm) were analyzed by means of the fluorescent dye 5,5,6,6-tetrachloro-1,1,3,3-tetraethylbenzimidazolcarbocyanine iodide (JC-1; Molecular Probes) as reported [42]. When live cells are incubated with JC-1, mitochondrial membrane polarization allows JC-1 to selectively enter mitochondria, which causes the reversible formation of JC-1 aggregates. JC-1 showed a spectral shift in emitted light from 530 nm (emission of JC-1 monomeric form; green) to 590 nm (emission of J-aggregate; green-orange) upon excitation at 488 nm. After 72 hours of serum deprivation control and PGRN deficient lymphoblasts were incubated with JC-1 at final concentration 5 mg/ml for 15 min at 37°C, in the dark. At the end of the incubation period, the cells were washed twice in cold PBS, resuspended in a total volume of 500 µl, and analyzed by flow cytometry. The ratio of red/green fluorescence intensity was calculated before and after 72 h of serum deprivation. A decrease in this ratio indicates mitochondrial depolarization (i.e loss of mithocondrial membrane potential).

### Immunoblotting Analysis

50–100 µg of protein from cell extracts were fractionated on a SDS polyacrylamide gel, and transferred to PVDF membrane (Bio-Rad). The amount of protein and the integrity of transfer were verified by staining with Ponceau-S solution (Sigma). The membranes were then blocked with non-fat milk and incubated, overnight at 4°C, with primary antibodies at the following dilutions: 1∶500 anti-pRb, 1∶1000 anti-CDK6, 1∶500 anti-p130, 1∶100 anti-cylin D1, 1∶200 anti-cyclin D2, 1∶500 anti-cyclin D3, 1∶200 anti-p16, 1∶100 anti-p18, 1∶5000 anti-β-actin, and 1∶1000 anti-lamin B1. The release of cytochome c from the mitochondria was assessed after cell fractioning to get cytosolic and crude mitochondrial extracts as described [43], using the ApoTrack™cytochrome c antibody cocktail. Signals from the primary antibodies were amplified using species-specific antisera conjugated with horseradish peroxidase (Sigma) and detected with a chemiluminiscent substrate detection system ELC (Amersham). The specificity of the antibodies was checked by omitting the corresponding primary antibody in the incubation medium. The relative protein levels were determined by scanning the bands with a GS-800 imaging densitometer provided with the Quantity One 4.3.1 software from BioRad, and normalized by those of β-actin.

### Statistical Analysis

Unless otherwise stated, all data represent means ± Standard Error of the Mean (SE). Statistical analysis was performed on the Data Desk package (version 4.0) for Macintosh. Statistical significance was estimated by analysis of variance (ANOVA) followed by the Fischer’s LSD test for multiple comparisons. Differences were considered significant at a level of p<0.05.
